# Pan-cancer analysis of NUDT21 and its effect on the proliferation of human head and neck squamous cell carcinoma

**DOI:** 10.18632/aging.205539

**Published:** 2024-02-12

**Authors:** Wenjing Liu, Yingna Pang, Xiaolu Yu, Doudou Lu, Yating Yang, Fandi Meng, Chengbi Xu, Ling Yuan, Yi Nan

**Affiliations:** 1 Key Laboratory of Ningxia Minority Medicine Modernization Ministry of Education, Ningxia Medical University, Yinchuan 750004, Ningxia Hui Autonomous Region, China; 2 Department of Otolaryngology Head and Neck Surgery, The Second Hospital of Jilin University, Changchun 130000, China; 3 College of Pharmacy, Ningxia Medical University, Yinchuan 750004, Ningxia Hui Autonomous Region, China; 4 Clinical Medical College, Ningxia Medical University, Yinchuan 750004, Ningxia Hui Autonomous Region, China; 5 Traditional Chinese Medicine College, Ningxia Medical University, Yinchuan 750004, Ningxia Hui Autonomous Region, China

**Keywords:** NUDT21, pan-cancer research, HHNSCC, immune microenvironment, apoptosis

## Abstract

Background: Based on bioinformatics research of NUDT21 in pan-cancer, we aimed to clarify the mechanism of NUDT21 in HHNC by experiment.

Methods: The correlation between differential expression of NUDT21 in pan-cancer and survival prognosis, genomic instability, tumor stemness, DNA repair, RNA methylation and with immune microenvironment were analyzed by the application of different pan-cancer analysis web databases. In addition, immunohistochemistry staining and genetic detection of NUDT21 in HHNCC tumor tissues by immunohistochemistry and qRT-PCR. Then, through *in vitro* cell experiments, NUDT21 was knocked down by lentivirus to detect the proliferation, cycle, apoptosis of FaDu and CNE-2Z cells, and finally by PathScan intracellular signaling array reagent to detect the apoptotic protein content.

Results: Based on the pan-cancer analysis, we found that elevated expression of NUDT21 in most cancers was significantly correlated with TMB, MSI, neoantigens and chromosomal ploidy, and in epigenetics, elevated NUDT21 expression was strongly associated with genomic stability, mismatch repair genes, tumor stemness, and RNA methylation. Based on immunosuppressive score, we found that NUDT21 plays an essential role in the immunosuppressive environment by suppressing immune checkpointing effect in most cancers. In addition, using HHNSCC as a study target, PCR and pathological detection of NUDT21 in tumor tissues was significantly increased than that in paracancerous normal tissues. *In vitro* cellular assays, silencing NUDT21 inhibited proliferation and promoted apoptosis in FaDu and CNE-2Z cells, and blocked the cell cycle in the G2/M phase. Therefore, the experiments confirmed that NUDT21 promotes the proliferation of FaDu by suppressing the expression of apoptotic.

## INTRODUCTION

Cancer, as the main cause of global morbidity and mortality, has brought huge health and economic burdens to society. Currently, cancer treatment strategies encompass surgical resection, radiotherapy, adjuvant chemotherapy, targeted therapy, and immunotherapy. While these approaches have yielded some results, patients still face unsatisfactory prognoses and quality of life due to toxic side effects and drug resistance [1]. Hence, it is crucial to urgently identify effective targets and novel tumor biomarkers that can aid in cancer prevention and treatment. The objective of pan-cancer research is to uncover disparities and resemblances in gene expression across different cancer types, thereby furnishing a theoretical basis for investigating specific genes in cancers [2].

Human head and neck carcinoma (HHNC) refer exclusively to cancers arising from the upper aerodigestive tract or more broadly to any malignancy originating in the head and neck region, and the main type is human head and neck squamous cell carcinoma (HHNSCC) [3, 4]. Globally, HHNSCC has been reported as the sixth leading cause of cancer. There are approximately 500,000 new cases of HHNSCC reported worldwide each year, and the incidence rate and mortality of this cancer have been steadily increasing in recent years [4]. In addition to high-risk human papillomavirus (HPV) infection, smoking and alcohol consumption also promote the development of these cancers. Due to its involvement in oropharyngeal tumor development, HHNSCC can be classified as HPV-negative or HPV-positive [5]. Currently, the main treatment strategies for HHNSCC include surgery, targeted therapy, radiotherapy, and immunotherapy. Although these treatments are successful in clinical practice, the prognosis and survival of patients are not ideal due to problems such as drug resistance and by-effect [1]. Therefore, there is a need to identify new effective therapeutic targets to improve the clinical management of HHNSCC patients.

NUDT21 (also known as CFIm25) is a member of the Nudix hydrolase family, which has a highly conserved subunit of the fission factor-Im complex (CFIm), which consists of four polypeptides (CFIm25, CFIm59, CFIm68 and CFIm72) and is involved in the assembly of eukaryotic pre-mRNAs [6]. This subunit possesses a NUDIX hydrolase domain and functions as a true RNA-binding protein [7]. The protein-RNA interaction is the first step in the assembly of the 3’-end processing complex, which facilitates the recruitment of additional processing factors [8]. Enable NUDT21 to influence the selection of APA sites by binding to proximal dissociation and alternative polyadenylation (APA) sites, and guide APA [9]. Therefore, as an APA-related protein, NUDT21 may participate in several leading regulatory processes [10]. The advantage of APA in tumor progression lies in the induction of oncogenes by shortening the 3’-UTR and inactivation of tumor suppressor genes through miRNA competitive rerouting [11]. The shortening of the 3’UTR on the mRNA leads to enhanced proliferation and tumorigenicity of cancer cells. According to reports, NUDT21 has dual carcinogenic and inhibitory effects in cancer [12]. Current studies have demonstrated that NUDT21 regulates bladder cancer [13], hepatocellular carcinoma [14], glioblastoma [15], leukemia [16], breast cancer [17], and gastric cancer [18]. Justin Brumbaugh identified NUDT21 as a novel post-transcriptional regulator of cell fate, and in alternative polyadenylation and established a direct link between chromatin signaling [19]. These findings identify a potential association between NUDT21 and cancer progression. However, the role of NUDT21 in HHNSCC and its underlying mechanism have not been fully elucidated.

**Figure 1:**
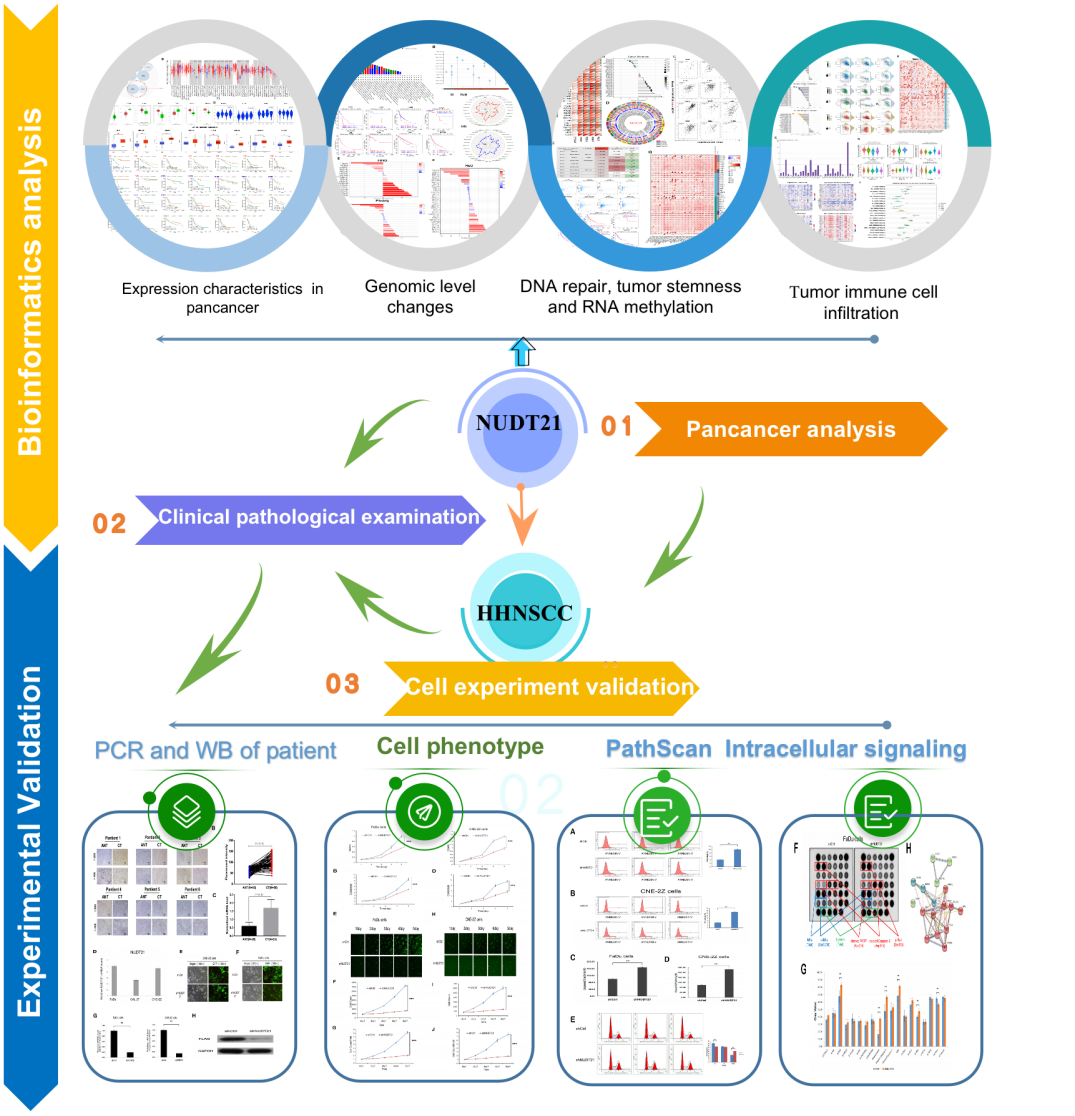
The workflow of this study.

The occurrence and development of tumors are multifactorial, involving the activation or inhibition of oncogenes, immune escape, chromosomal instability, epigenetic changes, and abnormal cellular signaling [20]. Currently, the mechanism of NUDT21 in tumor development remains unclear. Therefore, a comprehensive analysis of NUDT21 in various cancers was conducted through pan-cancer research. In particular, this study focused on HHNSCC as the research object to identify new effective therapeutic targets. The findings of this study provide new insights for the prevention and personalized treatment of HHNSCC. The schematic diagram illustrating the research design is presented in Figure 1.

## RESULTS

### Expression characteristics of NUDT21 in pan-cancer and its relationship with tumor prognosis

**Figure 2:**
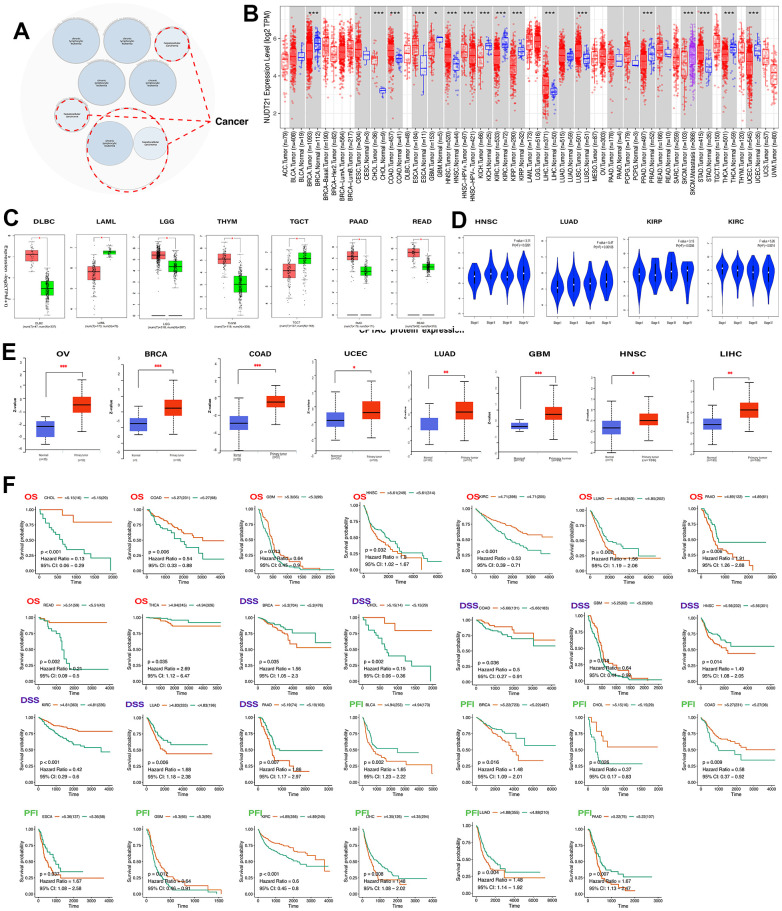
**Differential expression of NUDT21 and predicted survival in pan-cancer.** (**A**) Analysis of NUDT21 associated diseases using the Open Target web tool. The red dashed line indicates NUDT2 associated cancers. (**B**) Gene expression levels of NUDT21 in pan-cancerous tissues and its corresponding control tissues were analyzed using the TIMER2.0 method. Tumor and normal tissues are indicated in red and blue and SKCM metastatic tissues in purple, respectively. (**C**) Box plots of gene expression levels of NUDT21 in tumor and normal tissues of seven cancer types were plotted on GEPIA2.0. T and N represent tumor and normal tissues. (**D**) The expression levels of NUDT21 in four tumor types at four different stages were analyzed using the GEPIA2.0 method. (**E**) The UALCAN method was used to compare the protein expression differences of eight tumor tissues between normal and tumors tissues. (**F**) Kaplan-Meier curves were plotted to predict OS (red), DSS (blue) and PFI (green) in TCGA patients, * represents p<0.05, ** represents p<0.01, *** represents p<0.001.

The bubble map showed that NUDT21 was associated with diseases such as chronic lymphatic leukemia and hepatocellular carcinoma (Figure 2A). Subsequently, we analyzed the differences in NUDT21 mRNA expression levels between pan-cancer and corresponding normal tissues; NUDT21 gene was significantly changed in 16 tumor types, including significant upregulation of expression in CHOL, COAD, ESCA, HNSC, LIHC, LUSC and STAD, while in BRCA, GBM, KICH, KIRC, KIRP, PAAD, SKCUM, THCA, and UCEC were down-regulated in expression (Figure 2B). In tumors tissue, we compared the difference in NUDT21 mRNA expression on GEPIA2.0 and the difference in NUDT21 protein expression on UALCAN. According to the GEPIA2.0 results, which showed that NUDT21 mRNA was increased in DLBC, THYM, READ, LGG, PAAD. However, downregulated in LAML and TGCT (Figure 2C). In addition, NUDT21 was negatively correlated with elevated staging of HNSC, LUAD, and KIRP, and positively correlated with elevated staging of KIRC (Figure 2D). According to the UALCAN results, NUDT21 protein expression was upregulated in cancers of OV, BRCA, COAD, LUAD, LIHC, HNSC, UCEC, and GBM (Figure 2E). To understand the prognostic significance of NUDT21, we plotted Kaplan-Meier curves on TCGA data. We noted lower percentages of overall survival (OS) for NUDT21 with elevated expression of LUAD and PAAD, THCA; lower percentages of the disease-specific survival (DSS) for BRCA, HNSC, LUAD, and PAAD; and shorter progression-free intervals (PFI) for BLCA, BRCA, ESCA, LIHC, LUAD, and PAAD. In contrast, higher OS in CHOL, COAD, GBM, and KIRC was associated with higher DSS in CHOL, COAD, GBM and prolonged PFI in CHOL, COAD, GBM, and KIRC (Figure 2F). These results suggest that NUDT21 may be a cancer driver gene in HNSC and promotes BRCA progression, but it may be a protective gene in CHOL. The combined results showed that NUDT21 was extremely expressed and had a poor prognosis in LUAD, PAAD, HNSC and THCA, while it had a better prognosis in CHOL, COAD, GBM, and KIRC, demonstrating the heterogeneity of NUDT21 expression in different tumor type.

### Genomic level changes of NUDT21 gene in pan-cancer

**Figure 3:**
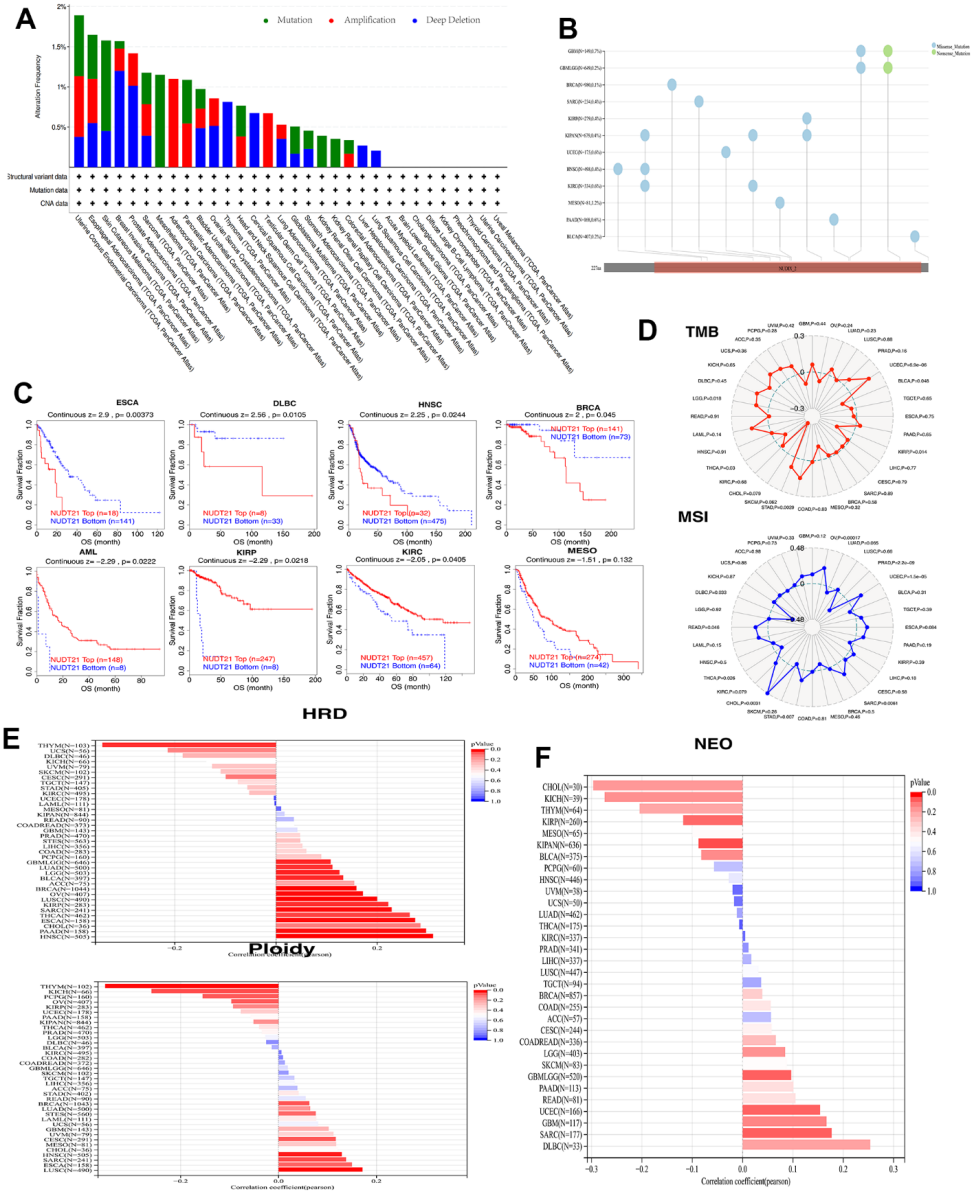
**NUDT21 has been linked to genomic instability in cancer.** (**A**) Genomic alterations of NUDT21 in TCGA pan-cancer were analyzed, including mutations, amplifications and deep deletions. (**B**) Landscape of SNV of NUDT21 in pan-cancer containing missense mutations, shift deletions and splice sites. (**C**) Kaplan-Meier plots using the TIDE web tool to show the prognostic significance of CNVs of NUDT21 in eight cancers. (**D**) Radar plot showing the association between TMB (top), MSI (bottom) and NUDT21 in patients with pan-cancer; dashed circles indicate a correlation coefficient of 0, and the intersection of solid lines (red or blue) inside the dashed circle indicates a negative correlation coefficient, and outside the circle indicates a positive correlation coefficient. (**E**) Bar graph showing the expression coefficients between HRD or ploidy and NUDT21 expression. (**F**) Bar graph shows the association between NUDT21 expression and neoantigen counts in pan-cancer.

Cancer is characterized by alterations in the genome. To find whether the NUDT21 mRNA is changed at the genomic level, we show the results of CNV and SNV analysis of NUDT21 pan-cancer, which revealed that NUDT21 in ACC, has a high amplification rate, a high mutation rate in MESO, and a high profound deletion rate >1% in BRCA, while no high SNV rate was observed (Figure 3A, 3B). Applying CNV levels to a subset of TIDE patients, increased expression of NUDT21 was connected with longer survival in KIRP, AML, KIRC and EMSO. However, lower survival in ESCA, DLBC, HNSC, and BRCA (Figure 3C). In addition, we performed analyses of MSI, TMB, neoantigens and ploidy correlations with NUDT21, as these genomic alterations are commonly seen in cancer and affect patient prognosis and treatment response. As shown in Figure 3D, NUDT21 was positively correlated with TMB in 3 tumor types (STAD, LGG, UCEC) and with MSI in tumor types (OV, UCEC, SARC, STAD, CHOL, READ). In contrast, there was a negative association with TMB in 3 cancers (BLCA, KIRP, THCA) and a negative association with MSI in 3 cancers (PRAD, THCA, DLBC). For HRD analysis, NUDT21 showed a significant negative correlation with HRD in THYM, followed by UCS, DLBC and UVM, and a positive correlation with HNSC, followed by PAAD and CHOL. For aneuploidy, NUDT21 was significantly negatively correlated in thymic carcinoma, KICH, PCPG and OV, while a negative correlation with four cancers (LUSC, ESCA, SARC, HNSC) were positively correlated (Figure 3E). As shown in Figure 3F, four cancers (CHOL, KIOH, THYM, KIRP) showed a negative correlation of neoantigen with NUDT21 expression and a positive correlation with DLBC, SARC, GBM, and UCEC. The above results firmly suggest that the NUDT21 gene is significantly correlated with TMB, MSI, neoantigens, and chromosomal ploidy in a variety of cancers, and that NUDT21 is a potential biomarker of genomic stability in THYM and HNSC.

### NUDT21 in relation to cancer DNA repair, tumor stemness and RNA methylation

**Figure 4:**
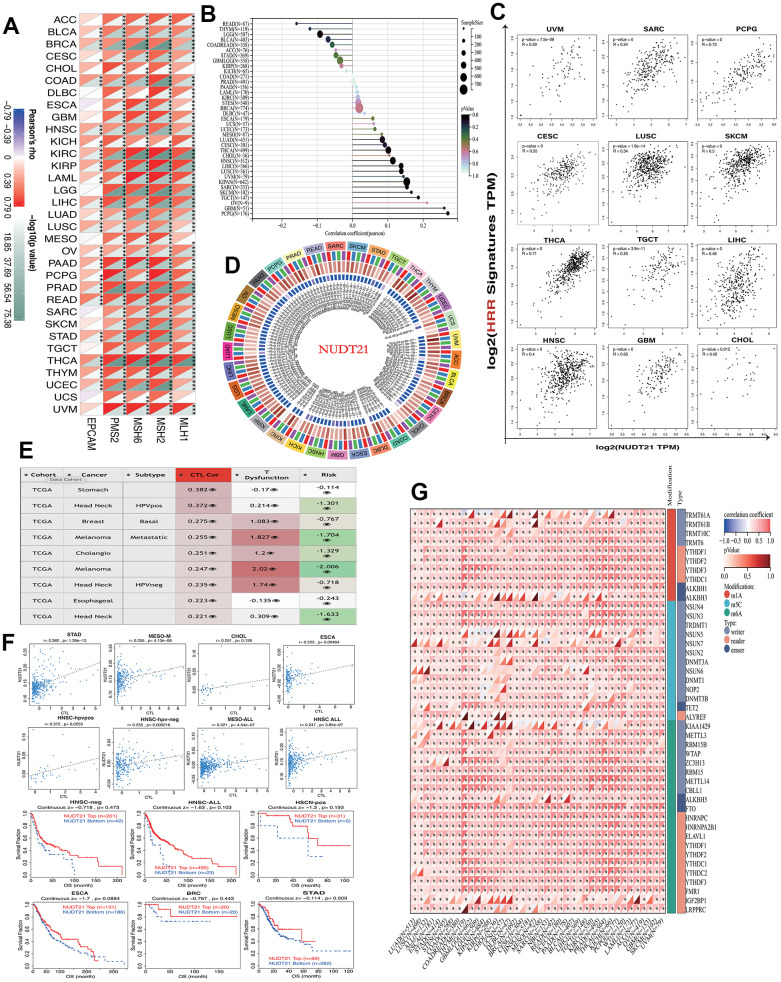
**NUDT21 is involved in cancer DNA repair, tumor stemness and epigenetic regulation.** (**A**) Heat map showing the association between NUDT21 and 5 MMR genes in pan-cancer. *, ** and *** represent p <0.05, p <0.01 and p <0.001, respectively. (**B**) Interrelationship between tumor stemness and NUDT21 expression in the lollipop plot, the size of the dots represents the sample size, while the color indicates the p-value. (**C**) Correlation scatter plots of 12 cancers showing the correlation between HRR features of 30 genes and NUDT21 expression. (**D**) Circos plot showing the correlation between four methyltransferases and NUDT21 expression. The first (outermost) circle refers to the name of the pan-cancer, and the second layer indicates the four sulfotransferases DNMT1, DNMT2, DNMT3A, and DNMT3B labeled in red, blue, green, and purple, respectively. The third layer shows green and brown representing negative and positive correlation coefficient values, respectively, and the innermost blue block indicates p-values (lower p-values correspond to dark blue). (**E**) The correlation between NUDT21 methylation levels and CTL correlates was retrieved in the methylation module of the TIDE web tool, the third column is the CTL correlation, and the fourth column is the CTL dysfunction z-score of the interaction term. (**F**) Association between NUDT21 methylation levels and CTL markers and between survival analysis of NUDT21 hypermethylated and hypomethylated subgroups in scatter plots and Kaplan-Meier plots. (**G**) Heat map showing the correlation between NUDT21 expression and RNA modifications in pan-cancer. * p< 0.05.

The stability of cancer genomes is mainly due to various DNA repair mechanisms, such as DNA-MMR and DNA-HRR, which also help in the preservation of cancer stem cells. Therefore, we analyzed the relationship between NUDT21 and MMR-associated genes, including EPCAM, MLH1, MSH2, MSH6, PMS2M, and HRR signatures with tumors. NUDT21 has been found to be positively associated with multiple MMRs in most cancers, such as HNSC, KICH, KIRC, KIRP, LAML, LIHC, LUAD, LUSC, OV, PADD, PRAD, READ, ATAD, THCA, THYM, and UCEC (Figure 4A). Regarding cancer stemness, we found that NUDT21 was strongly associated with PCPG, followed by positive correlations with GBM, CHOL, HNSC, LIHC, TGCT, THCA, SKCM, LUSC, CESC, PCPG, SARC, and UVM (Figure 4B). Not surprisingly, the 12 cancers with positive correlation between tumor stemness and NUDT21 also showed consistent HRR characteristics (Figure 4C), suggesting that NUDT21 interacts with cancer stemness through DNA repair. As DMPsi reflects DNA methylation in tumor, we investigated the effect of NUDT21 on epigenetic regulation in cancer. NUDT21 showed a significant positive correlation with methyltransferases in nearly all cancers (Figure 4D). Their strong positive correlation in cancer suggests that in most cancers, elevated NUDT21 promotes methylation of target gene promoters and represses their expression. We also examined the interaction between NUDT21 promoter methylation and cancer subtypes, CTL risk and TIDE. Nine major cancer subtypes positively associated with CTLs (Figure 4E); In HNSC, CHOL and MESO, ESCA, NUDT21 promoter methylation was positively correlated with CTL infiltration. The Figure 4F shows scatter plots and Kaplan-Meier curves associated with NUDT21, indicating that NUDT21 promoter methylation correlates with CTL infiltration, and elevated NUDT21 expression predicted prolonged survival in three HNSC subtypes and ESCA, BRCA, and STAD. In addition to DNA methylation, we further investigated the association of NUDT21 with RNA-regulated gene expression. Surprisingly, we found that elevated expression of NUDT21 correlated with RNA regulatory genes in numerous cancers, including numerous cancers gm1A, m5C and m6a (Figure 4G), suggesting that NUDT21 is also involved in RNA modification.

### Molecular mechanisms and related pathways involved in NUDT21

**Figure 5:**
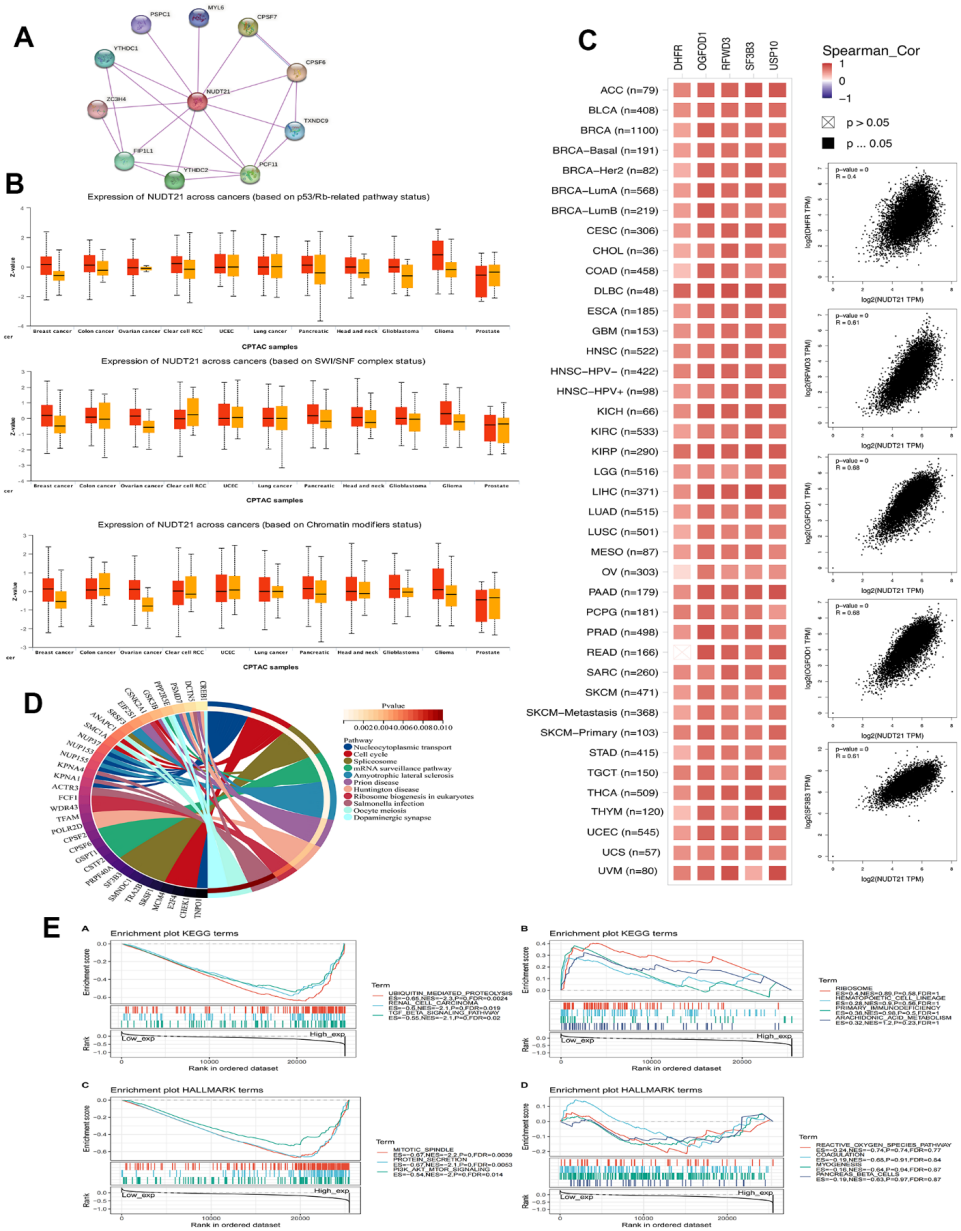
**NUDT21 is involved in chromatin remodeling, cancer immunity, and related pathways.** (**A**) PPI protein interactions network of NUDT21. (**B**) Box plots of NUDT21 expression between somatic alteration groups or non-alteration groups at the level of 11 cancer pathways were obtained by the UALCAN web tool. (**C**) Correlation between NUDT21 and the top 5 co-expressed genes in each cancer type (left) and in all cancer samples (right). Spearman_Cor indicates biased correlation. (**D**) STRING plots of the top 100 NUDT21 co-expressed genes identified on GEPIA2.0 enriched for GO pathways. Only the top genes of each pathway are listed at the left end of the corresponding ribbon. (**E**) Enrichment maps of KEGG and HALLMARK in pan-cancer were analyzed by GSEA, divided by median expression of NUDT21.

To study the functional roles of NUDT21 and its interacting or co-expressed genes in cancer, we performed a sequential functional enrichment analysis. Protein-protein interactions were validated from the STRING web tool, showing NUDT21 in relation to 10 protein-protein interactions (PPI) (Figure 5A). We then compare the expression of NUDT21 on UALCAN between the altered and unaltered pathways and note that NUDT21 was elevated in BRCA, HNSC, the altered SWI/SNF complex in Glioma, and the p53/rb related pathway (Figure 5B). Furthermore, the 100 most common genes co-expressed with NUDT21 were analyzed using GEPIA2.0, and the 5 most important genes (DHFR, OGFOD1, RFWD3, SF3B3, USP10) were associated with NUDT21 in most cases (Figure 5C). GO functional enrichment showed that NUDT2 was closely associated with nucleoplasmic transport and cell cycle activity was firmly correlated (Figure 5D). In addition, GO and KEGG results of GSEA showed that co-expressed genes of NUDT21 were closely associated with protein production secretion, hydrolysis, and TGF-β, PI3K-AKT related pathways (Figure 5E).

### NUDT21 participates in tumor immune cell infiltration and cytokine-mediated immune regulation

To analyze the immune role of NUDT21 in tumor progression, we counted the immunological score of NUDT21 in pan-cancer. The NUDT21 was negatively correlated with ESTIMATEScore and immune score, including GBM-LGG, LGG, LUSC, and TARGET-NB, TARGET-WT in TCGA tumors. Stromal cell infiltration in GBM-LGG, LGG, TARGET-NB, SARC, LUSC was negatively correlated with NUDT21 and was negatively correlated and positively correlated with KIPAN mixed kidney cancer (Supplementary Figure 1A). Cancers in which NUDT21 was inversely associated with immune levels were also inversely associated with most immune checkpoints, including LUSC, THYM, TGCT, and TARGET-NB (Supplementary Figure 1B). However, we noted that in numerous cancers, some markers were positively associated with NUDT21. In pan-cancer, HMGB1 had the highest positive correlation with NUDT21, followed by TLR4, CD276, and TNFRSF4. We also noted that in OV, UVM, and PADD, NUDT21 positively correlated with up to 24 immune checkpoint genes, suggesting that NUDT21 was involved in immune checkpoint effects. Next, we examined whether NUDT21 was expressed by TISIDB in different tumor immune subtypes. The histograms showed that NUDT21 was significantly associated with 10 cancer immune subtypes (Supplementary Figure 1C). The violin diagram showed that the main 6 cancers increased expression of NUDT21 in C4 subtypes of LGG, STAD, LUSC, and KIRC, suggesting that a negative correlation with lymphocyte function (Supplementary Figure 1D). In addition, we analyzed the NUDT21 and chemokine, receptor, and immune activator relationships, as shown in the heat map (Supplementary Figure 1E), NUDT21 was negatively correlated with several chemokines (CCL1, 2, 3, 4, and 5), numerous receptors, and immunostimulatory agents in pan-cancer. We also noted that hyperinflation of the NUDT21 promoter is positively associated with most immunostimulants, suggesting that NUDT21 expression affects chemokine-mediated immunostimulation of cancer. Finally, we compared the differences in NUDT21 expression in cancer cell lines before and after cytokine treatment using the TISMO online tool (Supplementary Figure 1F). We found that NUDT21 expression was decreased in three cell lines after TNFg treatment and in two cell lines after IFNb treatment. From multiple perspectives, these results suggest that NUDT21 is involved in creating an immunosuppressive environment in many cancers, probably through the inhibition of immunoregulatory functions and immune checkpoint effects.

### Correlation of NUDT21 with M2 macrophages and other immune-suppressed cells

To profoundly explore the role of NUDT21 in immune cells, we analyzed its expression at the level of immune cells. We first ran the CIBER-SORT algorithm to obtain the correlation of 22 immune cells with NUDT21. We found strong positive correlations between NUDT21 and M2 macrophages in HNSC, SKCM, TGCT, KIRC and LUSC, while NUDT21 was negatively correlated with CD8+ T and activated NK cells in many cancers. In addition, Tregs were negatively correlated with NUDT21 in a variety of cancers (Supplementary Figure 2A). Taking M2 macrophages as the research object, using different calculation methods on TIMER2.0 to analyze the correlation between the degree of immune infiltration and NUDT21 expression in the whole tumor, and correlations were found in COAD, LUSC and UVM (Supplementary Figure 2B). In addition to M2 macrophages, we looked for other immunosuppressive cells that are also involved. Using TIMER2.0 online tool, we showed the correlation between NUDT21 and CAFs, Tregs, and MDSCs, and NUDT21 is positively associated with at least two cell types in multiple cancers, such as MESO, CESC, LICH, OV, PAAD, READ, SARC, SKCM, and UCEC (Supplementary Figure 2C). In addition to this, their purity-adjusted correlations (Supplementary Figure 2D), the highest correlations with NUDT21 among CESC, LIHC, MESO, PAAD, READ, SKCM and UCEC were found for MDSCs and CAFs. Since CD8+T was the most influenced cell during immunosuppression. In addition, we studied the relationship between NUDT21 and CD8+ T cell using the 10 algorithms on TMER2.0 online tool. With a large negative correlation in HNSC, BRCA, KIRC and UCEC (Supplementary Figure 2E). Furthermore, the TIDE web tool identified a negative association with neuroblastoma and a positive association with T cell dysfunction in endometrial cancer. CTL was found to be negatively correlated with NUDT21 in endometrial cancer (Supplementary Figure 2F). Therefore, additional exploration of the relationship between NUDT21 and different pan-cancer immunosuppressive cells suggests that NUDT21 also plays a role in CAF, Tregs and MDSCs and suppresses anti-cancer immunity by targeting CTL.

### Upregulation of NUDT21 expression in HHNSCC tissues

**Figure 6:**
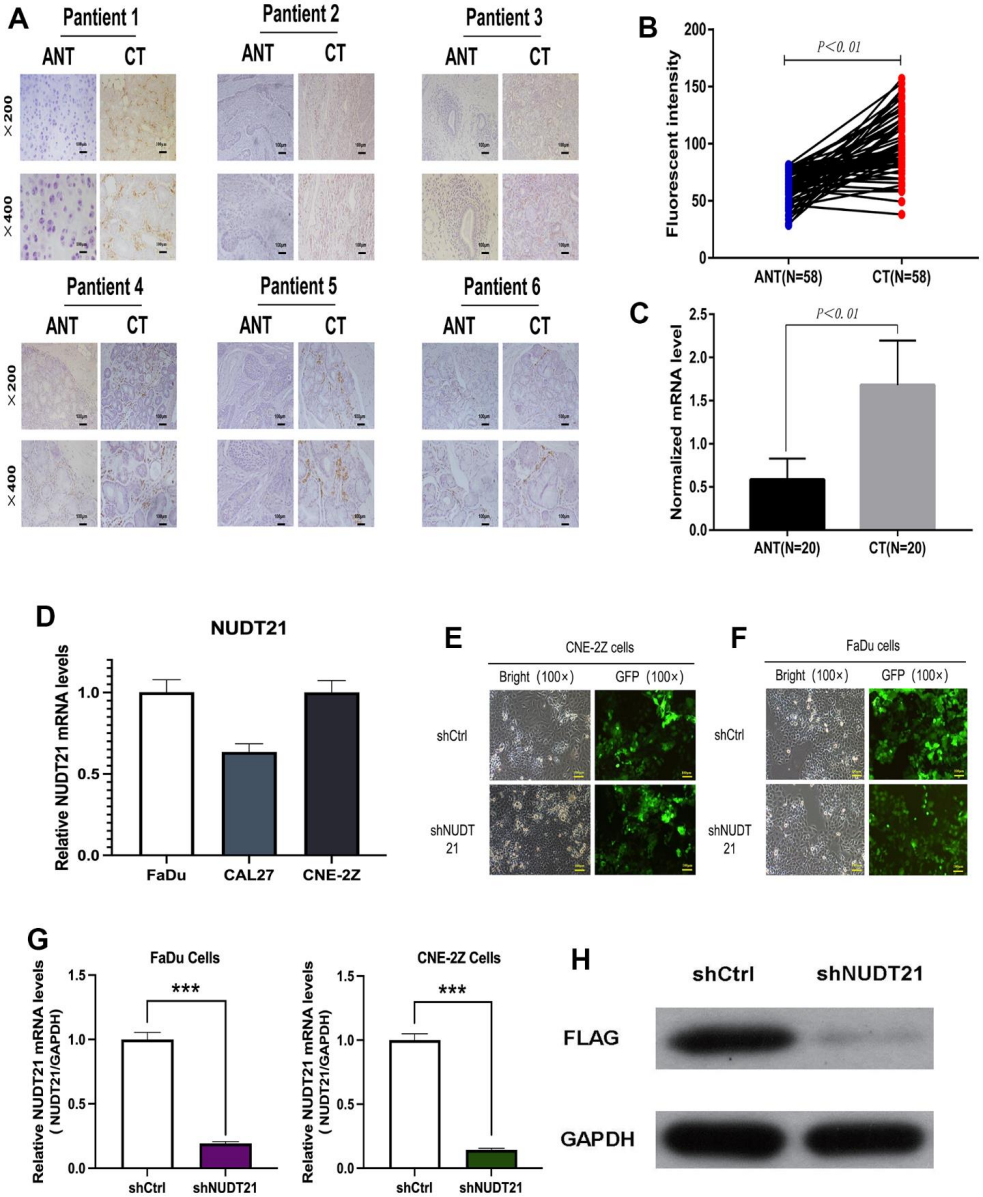
**Upregulation of NUDT21 expression levels in HHNSCC and knockdown of NUDT21 by lentivirus-mediated RNAi system.** (**A**–**C**) NUDT21 protein expression levels as well as gene levels in cancer tissues (CT) and paired adjacent non-tumor tissues (ANT) of 58 pairs of patients from the Second Hospital of Jilin University Cancer Center were counted by immunofluorescence method. Results included 58 pairs of patients (P<0.01) normalized with 18S rRNA. (**D**) Relative NUDT21 mRNA levels in three head and neck cancer cell lines (FaDu, CNE-2Z and CAL27). (**E**) Fluorescence photographs of FaDu and CNE-2Z cells infected by lentivirus (100×). (**F**, **G**) Identification of NUDT21 knockdown efficiency in FaDu and CNE-2Z cells by RT-PCR. (**H**) Identification of NUDT21 knockdown efficiency by FLAG protein blotting in FaDu cells. (shCtrl: control lentivirus; shNUDT21: lentivirus containing shRNA targeting NUDT21). **P <0.01 compared to shCtrl.

As confirmed by IHC staining and average optical density measurement (Figure 6A, 6B), NUDT21 was significantly expressed in cancer tissues compared to adjacent tissues in HHNSCC. Besides, the expression of NUDT21 mRNA was significantly higher in tumor tissues than in non-tumor tissues (Figure 6C).

### Lentivirus expressing NUDT21 shRNA was successfully constructed

To study the expression of NUDT21 in HHNSCC cells, NUDT21 mRNA expression levels in FaDu, CAL27 and CNE-2Z three cell lines detected by qRT-PCR. As shown in Figure 6D, NUDT21 was expressed in all three cell lines, with the highest level observed in FaDu cells, followed by CNE-2Z cells. Therefore, we chose FaDu and CNE-2Z cells for follow-up research to study the NUDT21 function in HHNSCC proliferation. To confirm the regulatory effect of NUDT21 on the proliferation of FaDu and CNE-2Z tumor cells, we screened if shNUDT21 could effectively silence the expression of NUDT21 (Lv-shNUDT21). A negative shRNA expressing lentiviral shCtrl (Lv-shCtrl) was used as a negative control. After 72h, over 80% cells were observed to express GFP in both cell lines, indicating successful lentiviral infection (Figure 6E, 6F). In order to further verify the knockout efficiency, we measured NUDT21 mRNA expression in FaDu and CNE-2Z cells by qRT-PCR. The research results confirmed that compared with the Lv-shCtrl group, the expression of NUDT21 mRNA in Lv-shNUDT21 infected cells was significantly inhibited in two cells (corresponding to 80.6% and 85.8% suppression in FaDu and CNE-2Z cells respectively, P<0.05, Figure 6G). Furthermore, comparing with Lv-shCtrl, FLAG protein blotting also identified NUDT21 was successfully knockdown in Lv-shNUDT21 group (Figure 6H). Our data suggested NUDT21 shRNA-expressing lentivirus was successfully transfected.

### Knocked down NUDT21 inhibits the proliferation of HHNSCC cells

**Figure 7:**
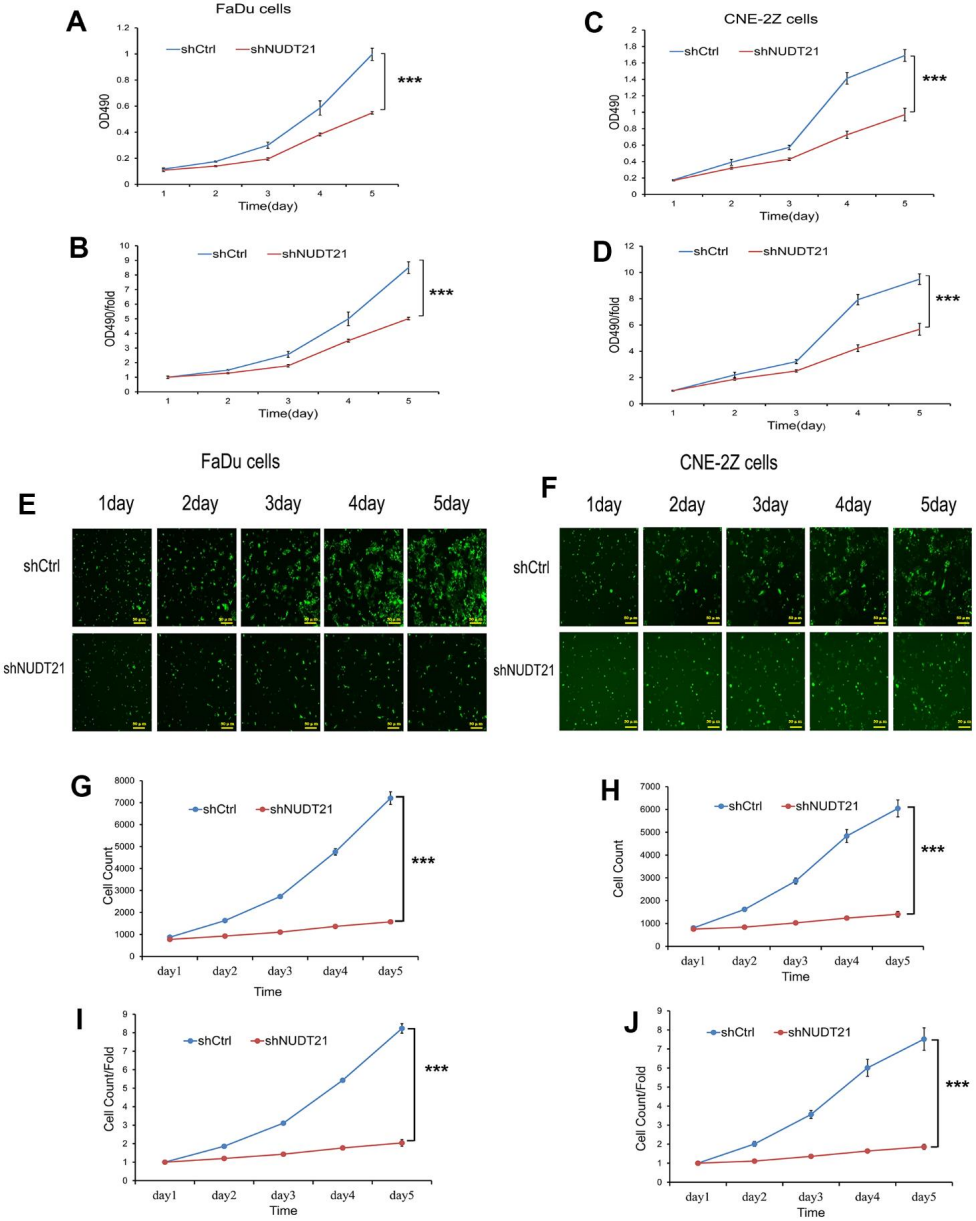
**Knockout of NUDT21 inhibits the proliferation of FaDu and CNE-2Z cells.** (**A**–**D**) Measurement of cell proliferation in shCtrl and shNUDT21 treated cells using MTT assay. (**E**, **F**) Raw images of shCtrl and shNUDT2-treated cell growth (unprocessed by software algorithms) were obtained by applying Celigo® cytometer imaging analysis. (**G**, **H**) shCtrl and shNUDT21-treated cells were inoculated in 96-well plates and cell growth was measured daily for 5 days. (**I**, **J**) Cell growth rates were monitored by assays on days 2, 3, 4, and 5. (shCtrl: control lentivirus; shNUDT21: lentivirus containing shRNA targeting NUDT21). ***P<0.001 compared to shCtrl.

In order to determine the effect of NUDT21 on the proliferation of HHNSCC cells, the OD value of cell proliferation was measured once a day by MTT method and Celigo method, and the images of cell growth were observed for 5 consecutive days. Our results showed that the proliferation rate of both Lv-shNUDT21-infected FaDu and CNE-2Z cells were lower compared with Lv-shCtrl-infected cells (P<0.01, Figure 7A–7J), which proved that knocking down NUDT21 significantly reduced the proliferation of FaDu and CNE-2Z cells.

### Knocked down NUDT21 enhances apoptosis of HHNSCC cells

**Figure 8:**
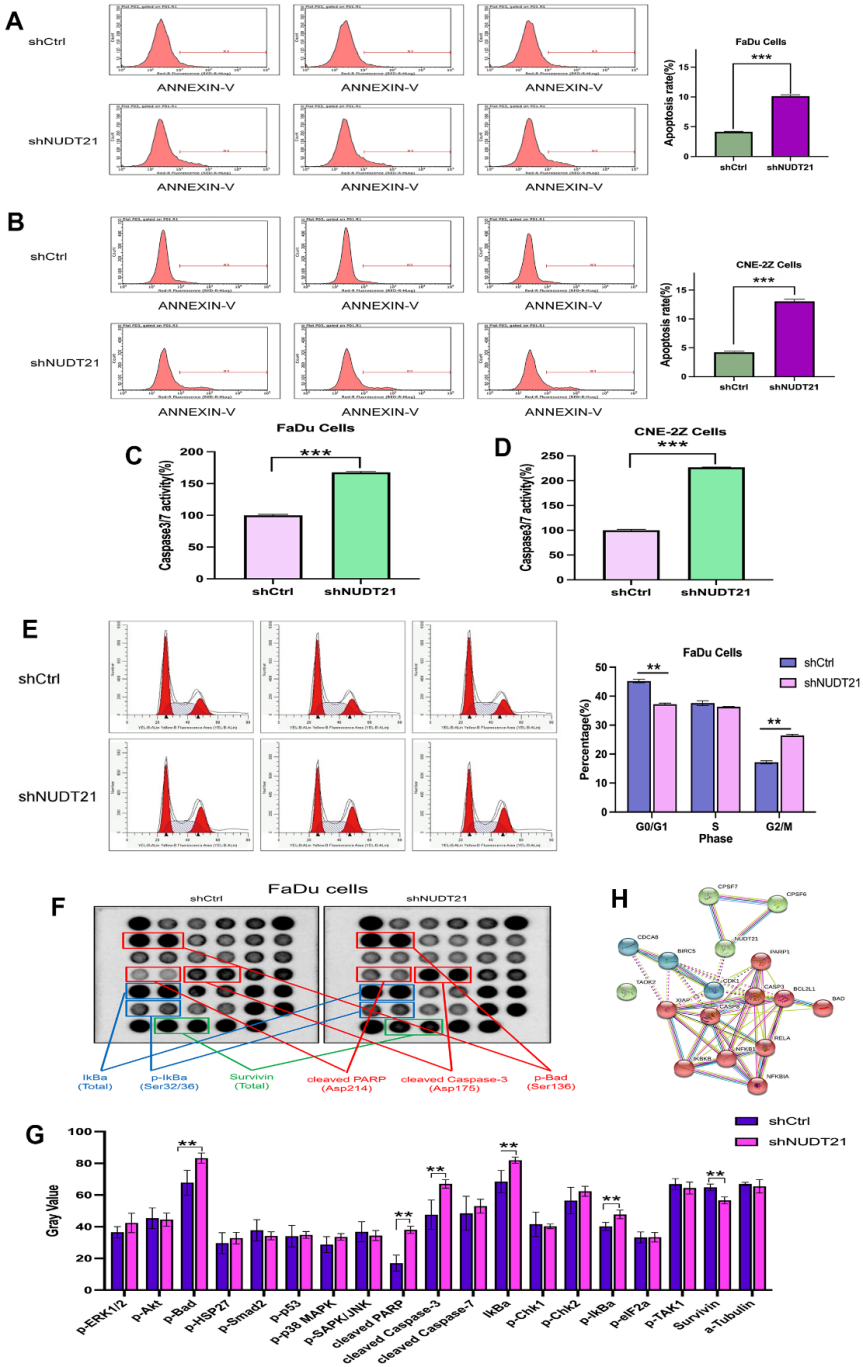
**Knockdown of NUDT21 increases apoptosis in FaDu and CNE-2Z cells and blocks the cell cycle of FaDu cells.** (**A**, **B**) Cell death was determined by Annexin-V staining and flow cytometry, and shNUDT21-treated cells showed a significant increase in apoptosis compared to shCtrl-treated cells. (**C**, **D**) Measurement of Caspase 3/7 activity. shNUDT21-treated cells showed a significant increase compared to shCtrl-treated cells. (**E**) Cell cycle analysis of FaDu cells by flow cytometry. shNUDT21-treated cells showed a significant decrease in G1 and a significant increase in G2/ M compared to shCtrl-treated cells. (shCtrl: control lentivirus; shNUDT21: lentivirus containing shRNA targeting NUDT21). **p<0.01 compared to shCtrl. (**F**, **G**) Modification of IkBa (Total), p-IkBa (Ser32/36), Survivin (Total), cleaved PARP, cleaved Caspase-3, p-Bad in shNUDT21-treated cells. (**H**) Network of NUDT21-related molecular proteins in cells. **p<0.01 compared to shCtrl.

To test whether NUDT21 expression affected the apoptosis of HHNSCC cells, we downregulated the expression of NUDT21 in each cell line. The Annexin V staining was used to detect cell apoptosis. As shown in Figure 8A, 8B, compared with Lv-shCtrl, Lv-shNUDT21 significantly increased the apoptosis of FaDu and CNE-2Z cells. (Lv-shCtrl: 4.16±0.07%, Lv-shNUDT21: 10.14±0.22%, P<0.001). We then measured the levels of Caspase 3/7 activity (Figure 8C, 8D). The results showed that the levels of Caspase 3/7 activity in FaDu and CNE-2Z cells were significantly higher in Lv-shNUDT21 compared to Lv-shCtrl (Lv-shCtrl: 100.00±1.59%, Lv-shNUDT21: 167.75±1.38%, P<0.01). The results suggested that NUDT21 expression is a key factor in the apoptosis of HHNSCC cells.

### Knocked down NUDT21 interrupts the cell cycle in G2/M phase of HNSCC cells

To understand the underlying mechanism that suppresses cell proliferation, the cell cycle changes were measured using flow cytometry. We observed the percentage of cells in different cell cycles (G1 phase, S phase, G2/M phase) as shown in Figure 8E. Compared to Lv-shCtrl (G1: 45.22±0.59%, S: 37.58±0.86%, G2/M: 17.19±0.50%), the percentage of cells in Lv-shNUDT21-infected FaDu cells was reduced in the G1 phase (37.25±0.38%, P<0.001), while not significant difference in the S phase (36.31± 0.13%, P>0.05) and increased in G2/M phase (26.44±0.44%, P<0.001), indicating cell cycle arrest at G2/M. These results suggest that NUDT21 knockdown inhibits cell proliferation by inducing cell cycle arrest at G2/M phase.

### Knocked down NUDT21 affects the biological mechanism of HHNSCC cells

The STRING database predicted the relationship between NUDT21 and cell apoptosis or cycle related proteins (CDK1, CASP8, CASP3, BCL2L1, BAD, PARP1, and IKBA, etc.) (Figure 8H). The protein interaction network diagram of NUDT21 suggests its potential role in regulating the HNNSCC cell proliferation mechanism by influencing apoptosis-related proteins. To further elucidate the regulatory mechanisms of NUDT21 in HHNSCC tumorigenesis, multiple signaling pathways in FaDu cells were analyzed after NUDT21 knockdown. NUDT21 triggered signaling was identified using the PathScan intracellular signaling array kit. Knockdown of NUDT21 significantly inhibited the activation of SURVIVIN (Total, 12.64%, P=0.0001) and induced the activation of PARP (Asp214,123.92%, P=0.0000), CASP-3 (Asp175, 40.69%, P=0.0028), IKBA (Total, 19.67%, P=0.0005) activation and significantly increased phosphorylation of BAD (Ser136, 22.89%, P=0.0012), IKBA (Ser32/36, 18.77%, P=0.0043), indicating that SURVIVIN, PARP, CASP-3, IKBA and BAD play an important role in NUDT21-induced cell cycle arrest and apoptosis play an important role in NUDT21-induced cell cycle arrest and apoptosis (Figure 8F, 8G).

## DISCUSSION

In our study, we first systematically described the differential expression of NUDT21 and its prognostic value in pan-cancer analysis through bioinformatics research. The prognosis of NUDT21 in pan-cancer and its role in the immune microenvironment were investigated by different online databases. Pan-cancer results show that potential correlations of NUDT21 expression with TMB, MSI, DNA repair, RNA methylation, levels of immune infiltration, and various immune-related genes have been assessed in various cancers. In addition to this, the biological functions and pathways of NUDT21 were also examined using KEGG and gene set enrichment analysis (GSEA). Furthermore, the results revealed that upregulation of NUDT21 expression suppresses immunostimulatory functions and immune checkpoint effects. Thus, NUDT21 plays an important role in creating an immunosuppressive environment for a variety of cancers. In certain cancers, NUDT21 upregulation was correlated with increased infiltration of immune cells such as M2 macrophages. Pan-cancer-based analysis provides insight into the function of NUDT21 in the tumorigenesis of different cancers as well as providing a pre-study basis for the study of some cancers. Notably, in HNSCC cancers, elevated expression of NUDT21 was significantly associated with poor prognosis. Subsequently, considering the pathological examination of clinical patients’ HHNSCC tissue specimens, we have chosen HHNSCC as the experimental subject for this research. We confirmed for the first time that the expression of NUDT21 was significantly upregulated in HNSCC patient and in HHNSCC cells by PCR and pathological comparisons, which showed that NUDT21 was significantly increased in cancer tissues compared with adjacent normal tissues. Silencing of NUDT21 inhibited the proliferation and promoted apoptosis of FaDu and CNE-2Z cells and blocked the cell cycle in the G2/M phase. Additional experiments confirmed that NUDT21 promotes the proliferation of FaDu by regulating the expression of apoptosis pathway effectors (SURVIVIN, PARP, CASPASE-3, IKBA and BAD). Therefore, in this study, we confirmed that the HNNSCC biomarker NUDT21 promotes tumor development by inhibiting apoptosis.

Tumor occurrence is a complex process characterized by multiple steps and levels. Currently, inducing apoptosis in tumor cells is a key objective in cancer treatments. However, the presence of oncogene alterations and disturbances in the tumor microenvironment (TME) greatly impedes the occurrence of cell apoptosis. In the present study, we observed an upregulation of NUDT21, and it had a significant alteration of proteins related to the apoptotic pathway in FaDu and CNE-2Z. Analysis of the total levels and phosphorylation levels of the related signaling pathway proteins revealed that the upregulation of Caspase-3, which plays an important role in the progression stage of apoptosis, implies that apoptosis has occurred. On the other hand, an increase in phosphorylated BAD (apoptosis-promoting protein) and a decrease in SURVIVIN (apoptosis-inhibiting protein) indicated a further exacerbation of the apoptosis phenotype. Although severely damaged cells can be programmed to die by apoptosis, the DNA structure of those cells that are slightly damaged can be repaired by blocking some cell cycles. Thus, a significant increase in PARP associated with the base excision repair (BER) pathway could explain the DNA repair phenotype. Otherwise, increased levels of total IKBA and phosphorylation levels suggest that activated NF-κB stimulates the inflammatory response (cancer promoter). In different organ tumor studies, Zhu [21] demonstrated that reducing the protein level of NUDT21 in osteosarcoma tissue can inhibit cell proliferation and promote cell apoptosis. Zhang [22] found that NUDT21 was upregulated in chronic myelogenous leukemia, and the inactivation of NUDT21 inhibited proliferation and promoted apoptosis of K562 cells. Zheng [23] found that NUDT21 was a valuable marker in the prognosis of pancreatic ductal adenocarcinoma and can promote tumor proliferation and inhibit apoptosis through EIF2 signaling. Zhu [18] proved that the significant expression of NUDT21 in GC cells promotes tumor cell growth and metastasis by upregulating SGP2 in nude mice. Lou [24] demonstrated that the NF-kB inhibitor zeta (NFKBIZ) was a downstream target of NUDT21, and that MES recognition genes CHI3L1 and FN1 were differentially regulated in glioblastoma cells. In conclusion, the above findings confirmed that NUDT21 plays an essential role in tumor cell proliferation and apoptosis. And in this experiment, we also confirmed that the mechanism of NUDT21 action on HHNSCC is to promote proliferation by inhibiting the apoptotic pathway.

It is worth noting that the tumor microenvironment creates a long-term stress environment in cancer cells [25]. Preclinical data suggest that HHNSCC is a severe immunosuppressive disease characterized by abnormal secretion of pro-inflammatory cytokines and immune effector cell dysfunction [26]. The complex interactions of various immune checkpoints and stimulation pathways in HNSCC-TME occur in different cellular environments, with an important component being the infiltration of immunosuppressive cell populations [27]. In addition to direct suppression of immune effector cells, HNSCC regulates and recruits additional immune populations capable of modulating T and NK cell responses, including Tregs, myeloid-derived suppressor cells (MDSC), tumor-associated macrophages (TAM), and cancer-associated fibroblasts (CAF). Immunomodulation produced by these diverse cell populations cause the promotion of the tumor microenvironment. In addition, related literature found that NUDT21 recognizes tumor cell proliferation and immune cell infiltration of TME through events with APA [28], and this TME infiltration leads to the activation of NUDT21, which induces a different transcriptional program to address these changes. From this, we suggest that NUDT21 is closely related to the immune response in HNSCC. Furthermore, our study is based on pan-cancer research and reveals the important contribution of NUDT21 in the immunosuppressive environment of tumor by inhibiting immune stimulation function and immune checkpoint effects. Although traditional apoptosis is not immunogenic, several researches have shown that in certain conditions under Caspase deficiency, immunogenic apoptosis can absolutely be induced through activation of NF-κB signaling pathway [29] and cGAS/STING pathway [30] to trigger adaptive antitumor or antiviral immune responses. In the tumor microenvironment, apoptosis induced by drugs or cytotoxic immune cells is considered to be the main pathway for tumor cell elimination in the TME. Furthermore, the immunosuppressive properties of the TME (hypoxia, low pH, ROS) mediate the reduction and apoptosis of cytotoxic immune cells, thereby promoting the proliferation of tumor-promoting immunosuppressive cells such as Tregs, M2 macrophages, and myeloid suppressor cells (MDSCs) [31, 32]. Thus, tumor cell apoptosis in the TME is often impaired due to tumor cytotoxic immunity or loss of tumor cell apoptosis signaling [33]. Therefore, a major goal of cancer research is to initiate cancer cell-specific apoptosis in the TME [34]. In the present study, we suggest that silencing NUDT21 promotes the occurrence of a tumor immune response to HHNSCC, which leads to the development of immunogenic apoptosis.

## CONCLUSIONS

Therefore, in this study, we aimed to investigate the diagnostic efficacy and prognostic value of NUDT21 in cancer through comprehensive analysis tools. Subsequently, employing bioinformatics pan-cancer analysis and conducting functional experiments on HHNSCC cells, we discovered that NUDT21 functions as an oncogene in HHNSCC, promoting cancer cell proliferation, suppressing apoptosis, and playing a crucial role in the tumor microenvironment. Based on these findings, we propose that NUDT21 has the potential to serve as a diagnostic and prognostic biomarker for HHNSCC and could be targeted in future therapeutic interventions promoting personalized cancer treatment. However, due to time and cost constraints, this study only conducted cellular functional experiments and has not yet conducted in-depth research on the relationship between NUDT21 and the tumor microenvironment. Therefore, we plan to further investigate the role of NUDT21 in the immune regulation of HHNSCC in the tumor microenvironment. We will explore the activation, inhibition, or regulatory effects of NUDT21 on immune cells, and investigate its potential molecular mechanisms in HHNSCC immune escape, providing a theoretical basis for finding new immunotherapy strategies. Furthermore, we also intend to further validate the reliability of NUDT21 as a diagnostic and prognostic indicator for HHNSCC. To achieve this, we will expand the sample size, collect more clinical data, and conduct multicenter clinical studies. By doing so, we aim to verify the reproducibility and generalizability of our results, thus advancing the clinical application of NUDT21 as a potential therapeutic target.

## MATERIALS AND METHODS

### Bioinformatics analysis

#### Differential expression of NUDT21 and predicted survival in pan-cancer

Firstly, NUDT21-related diseases and disease phenotypes were searched through the genomic database Open Target Platform (https://platform.opentargets.org/). Differences in NUDT21 mRNA gene expression between cancer tissues and normal tissues were analyzed using the online tool TIMER2.0 (http://timer.cistrome.org/). TCGA and GTEx data from the GEPIA2.0 online tool were used to analyze gene expression in tumor types lacking sufficient normal samples, and NUDT21 mRNA expression in tumor staging. To compare NUDT21 protein expression in tumor tissue and normal tissue, we used the UALCAN Canceromics database (http://ualcan.path.uab.edu/); The NUDT21 expression and survival were analyzed through using the Kaplan-Meier (https://kmplot.com/).

#### Analysis of tumor-associated genomic alterations and antigenic correlations in NUDT21

The Cancer Type Summary module of cBioPortal (http://www.cbioportal.org/), a multidimensional cancer genomics database, was used to analyze the types (mutations, amplifications and profound deletions) and frequencies of three genomic alterations in pan-cancer, and the Mutations' module to obtain the distribution of single nucleotide variants of NUDT21 in pan-cancer, including missense, mutations, metastatic deletions and splice sites; the prognostic significance of NUDT21 Copy Numbers (CNVs) for survival is investigated in the Tumor Immune Dysfunction and Exclusion (TIDE) database Copy Number module (http://tide.dfci.harvard.edu). The correlations between tumor mutational burden (TMB), microsatellite instability (MSI), homologous recombination defect (HRD), ploidy, and neoantigen count and NUDT21 expression were analyzed using the SangerBox data analysis platform (http://past20.Sangerbox.com/Index) Genomic Heterogeneity and Gene expression Analysis module.

#### Analysis of NUDT21 correlation with tumor stemness, DNA mismatch repair, and epigenetic modification

Correlation analysis between NUDT21 and five mismatch repair genes (MMR) MLHI, MSH2, MSH6, PMS2, EPCAM in patients with pancreatic cancer; the correlation between tumor stemness and NUDT21 expression was analyzed using the tumor stemness and gene expression analysis module in the data analysis platform SangerBox (http://past20.Sangerbox.com/Index). The correlation between tumor stemness and NUDT21 expression was analyzed using the tumor stemness and gene expression analysis module in the SangerBox data analysis platform; 30 HRR (homologous recombination repair gene) genes were obtained in the ARIEL3 clinical trial, then entered into GEPIA2.0 to analyze the correlation between their expression and NUDT21; correlation between expression of four DNA methyltransferases (DNMT1, DNMT2, DNMT3A, DNMT3B) and NUDT21 in pan-cancer tissues; relationship between NUDT21 promoter methylation and cytotoxic lymphocytes (CTL) and patient survival using the TIDE methylation module; using the SangerBox data analysis platform, selecting the RNA modification gene analysis module in the pan-cancer study, plotting a heat map showing the relationship between NUDT21 and correlation of the modifier genes n1-adenosine (m1A), 5-methylcytosine (m5C) and n6-methyladenosine (m6A).

#### NUDT21 protein interaction network and functional enrichment analysis

The currently known experimentally validated protein-protein interaction networks for NUDT21 were retrieved using the STRING online web tool (https://www.string-db.org/); the pathway level of somatic changes in pan-cancer was retrieved in the UALCAN canceromics database (http://ualcan.path.uab.edu/) group and end-altered group between NUDT21 expression changes and analyze the correlation between the top 5 NUDT21 co-expressed genes in pan-cancer using TIMER2.0 and GEPIA2.0 though scatter plot to visualize; in addition, GEPIA2.0 analyzes the top 100 NUDT21 pan-cancer co-expressed genes enriched in GO pathway and uses SangerBox data analysis platform’s enrichment analysis circle mapping tool for visualization; enrichment maps of KEGG and HALLMAPK in pan-cancerous tissues by the GSEA easy analysis and visualization tool on this online platform.

#### Study of the immune role of NUDT21 in the tumor microenvironment

The ESTIMATEScore, ImmuneScore, StromalScore correlation analysis of NUDT21 in tumor tissues was analyzed using Sangerbox3.0 online tool (http://sangerbox.com/home.html), and the top 6 cancers in terms of Score correlation were represented by density plots; Sangerbox 3.0 online tool Gene Immune Analysis module correlation analysis of pan-cancer immune checkpoints and NUDT21 expression; comparison of expression levels of tumor subtype modules in pan-cancer immune subtypes using the immune analysis tool TISDB (http://cis.hku.hk/TISID/) in the top 6 cancer immune subtypes with NUDT21 expression. In addition, the expression of NUDT21, the association between chemokines, receptors, and immune stimulators were analyzed in the immunomodulators and chemokines modules, as well as the association between NUDT21 promoter methylation levels and immunostimulants. Using TISMO (http://tismo.Cistrome.org), a mouse homozygous tumor database for immunotherapy response and tumor immunity models, the expression levels of the NUDT21 in tumor cell lines before and after cytokine treatment were compared.

#### Immunosuppressive role of NUDT21 in the tumor microenvironment TIMER2.0

TIMER2.0 analyzed the immune cell infiltration analysis of NUDT21 in pan-cancer, as well as the analysis of M2 phagocyte infiltration by multiple algorithms; Furthermore, we examined the correlation between NUDT21 expression and the cell of Treg, CAFs and MDSCs expressed in multiple immune algorithms, as well as the correlation between NUDT21 and purity and purity adjustment among the three cells. CD8+ T cells correlated with NUDT21 in different cancers through different immune algorithms. In addition, we explored the relevance of NUDT21 expression in CTL cell dysfunction and CTL-correlated prognosis in the TIDE database.

### Experimental part

#### Clinical relevance study

We archived a total of 58 HHNSCC cancer samples, and these tumors were histologically and clinically diagnosed at the Second Hospital of Jilin University Cancer Center from September 2019 to March 2021. A total of 26 men and 32 women (mean age 62.8 ± 12.4 years) were identified. Tumors and paired normal tissues (at least 5 cm removed from the tumor and confirmed as normal by histological examination) were obtained from newly diagnosed patients and cancer patients who did not receive any treatment prior to sample collection. All patients taking part in the study provided written informed consent to the Ethics Committee of Jilin University before the start of the study.

#### Immunohistochemistry (IHC)

All surgically resected specimens were formalin-fixed and paraffin-embedded for histological examination. Immunohistochemistry (IHC) was performed on 4mm paraffin sections using NUDT21 antibody (Abcam, Cambridge, UK) as described in a previous report, stained sections were evaluated at 200X magnification and analyzed for mean optical density (MOD) of nine representative stained areas containing >90% tumor cells in each section. Here, color is measured in terms of the signal strength of positive pixels. The difference between the MOD data and the average MOD between the two groups was analyzed and compared by the T test, and P<0.05 was considered significant. IHC staining was quantified using AxioVision version 4.6 computerized image analysis system and an automated measurement program (Carl Zeiss, Jena, Germany).

#### Cell culture

The laryngeal cancer cell line FaDu, tongue squamous carcinoma cell line CAL27 and nasopharyngeal cancer cell line CNE-2Z were obtained from GeneChem (Shanghai, China). Cells were cultured at 37° C in Dulbecco’s Modified Eagle’s Medium (DMEM, Gibco) supplemented with 10% heat-inactivated fetal bovine serum (FBS) and 1% penicillin/streptomycin. Grow in 5% CO2 and keep in a humid environment.

#### Extraction of total RNA and RT-qPCR

Total RNA was extracted from cultured cells after 48 hours transfection and 20 patient samples (tumor and paired normal tissues) using Trizol solution (Invitrogen, USA), respectively. Reverse transcription synthesis of first stranded complementary DNA using PrimeScript RT Master Mix Perfect real-time software (TaKaRa, Shiga) from extracted total RNA. Detection of NUDT21 mRNA expression by RT-PCR using SYBR Premix Ex Taq on a 2720 Thermocycler Real-Time System was completed in real time (TaKaRa, Japan).

NUDT21: Forward, 5’-GGTTCACTCAGTTCGGCAACAA-3’

Reverse, 5’-CTCATGCGCTGAAATCTGGC-3’

GAPDH: Forward, 5’-TGACTTCAACAGCGACACCCA-3’

Reverse, 5’-CACCCTGTGCTGTAGCCAAA-3’

Each experiment was repeated three times using the formula 2 ^– ΔCT^ (CT; cycle threshold, ΔCT = CT (Target gene) - CT(GAPDH)).

#### Short hairpin RNA transfection

Short hairpin RNA sequences against NUDT21 (sh-NUDT21) and Nontarget control shRNA (sh-Ctrl) were designed and generated by Genepharma Co., Ltd. (Shanghai, China), as listed in Table 1. FaDu cells and CNE-2Z cells were plated at 1×10^6^ cells per well in a 6-well plate the day before transfection. The shRNAs (MOI=10) were transfected into the cells using Lipofectamine 2000 (Invitrogen, USA) according to the manufacturer’s instructions, after incubating for an additional 24 h at 37° C in a CO2 incubator. Two hours before transfection, the medium was changed to DMEM without FBS. This step helps improve transfection efficiency. Lentiviruses carry a green fluorescent protein (GFP) reporter gene driven by a cytomegalovirus promoter. GFP-expressing cells were counted by fluorescence microscopy at 72 hours post-infection to determine viral load. The cells were harvested to analyze the expression of genes of interest using Western blot assays and quantitative reverse-transcription PCR (qRT-PCR).

**Table 1:** Sequences of short hairpin RNA to NUDT21 genes.

**Gene name**	**shRNA sequences of NUDT21**
NUDT21(Target)	5′-ACCTCCTCAGTATCCATAT-3′
sh-Control	5′-TTCTCCGAACGTGTCACGT-3′
sh-NUDT21	5′-CCGGGAACCTCCTCAGTATCCATATCTCGAGATATGGATACTGAGGAGGTTCTTTTTG-3’

### Western blot

The total protein concentration was determined using the BCA protein assay kit. The protein sample (40 μg) was added to SDS-PAGE and electrophoresed at 50 V for 3 hours, and then transferred to PVDF membrane (Millipore, USA). After blocking with TBST containing 5% (w/v) nonfat dry milk for 1 hour at room temperature, anti-FLAG (Abcam, USA, #ab49763, diluted 1:2000) or mouse anti-GAPDH (Abcam, USA, #ab8245, dilution 1:2000) was incubated overnight or 4h. After washing with TBST, the membrane was incubated with HRP (horseradish peroxidase)-labelled goat anti-mouse IgG (Abcam, USA, #ab6789, dilution 1:2000) secondary antibody at room temperature for 2h. Membranes were analyzed using Ultra ECL detection reagent (Shanghai Yisen Biotechnology Co., Ltd., China).

### Cell viability assay

Briefly, groups of FaDu and CNE-2Z cells (Lv-shNUDT21, Lv-shCtrl) were vaccinated on 96-well plates at a density of 2×10^3^ cells per well. At the specified time point, 20 μg of MTT and DMSO was added to each well for 4 hours to make a final concentration of 5mg/ml. The reaction was then terminated by the addition of acidic isopropanol [10%SDS, 5%(v/v) isopropanol, and 0.01 mol/L HCl], and the wavelength of cell viability was extracted using an enzyme-linked immunosorbent assay (Bio-Rad, USA) by absorbance calculate.

### Celigo® cytometer imaging assay

FaDu and CNE-2Z cells infected with lentiviral vectors (shNUDT21 or shCtrl) were seeded in 96-well plates at a density of 2×10^5^ cells per well. Two types of green fluorescent cells were detected using a Celigo® cytometer (Nexcelom, Inc., USA). We accurately counted the number of green fluorescent cells per scan well by adjusting the assay setup input parameters. The ratio of the cell count at each time point of each group to the cell count at the first day of this group was calculated to obtain the cell proliferation multiple at each time point of this group, and growth curves based on cell proliferation folds were plotted according to cell proliferation folds and time points. We observed the control group and NUDT21 siRNA cell number for 5 days, calculated and analyzed the cell number, and constructed the cell proliferation curve.

### Flow cytometry

Apoptosis cells were detected using AnnexinV-APC staining, followed by flow cytometry analysis to analyze and detect cell apoptosis rate. For apoptosis analysis, 200 μl 1x binding buffer (Yeasen Biotech Co., Ltd., China) containing 10 μl Annexin V-APC was used for dark staining at room temperature for 15 minutes. Subsequently, we analyzed stained cells using flow cytometry. All experiments were repeated three times.

### Caspase 3/7 activity assay

The Caspase Assay System was utilized to detect DEVDase (Caspase 3/7) activity. FaDu cells were treated with 1 μm STS and lysed in lysis buffer from the Caspase-Glo®3/7 Assay kit (Thermo Fisher Scientific, USA). Protein content was determined and lysates incubated with Ac-DEVD-pNA for 4 h at room temperature. The reaction product was detected at 405 nm using a Bio-1200-II-A2 microplate reader (Thermo Fisher Scientific, USA). Each experiment was repeated three times.

### Cell cycle detection

On the 10th day, cells transfected with siRNA-NUDT21 Lentivirus or Ctrl Lentivirus were inoculated into 6-well plates in triplicate and incubated at 37° C for 5 days. After the cells were collected, they were washed twice with phosphate buffered saline (PBS), fixed with 75% pre-cooled ethanol at room temperature for 4 hours or overnight, and then stained with propidium iodide (PI, 50 mg/ml). Finally, the cell cycle of each cell group was analyzed using flow cytometry. Each experiment was repeated three times. The PI fluorescence intensity detected by the flow cytometer directly reflects the distribution state of DNA in each phase of the cell, thereby calculating the percentage of each phase.

### Intracellular signaling analysis

Phosphorylation and proteolysis are two ubiquitous post-translational covalent modifications and important regulatory mechanisms in biology. Detecting these changes in many cellular proteins has well-defined roles in cell biology and can provide broad insights into intracellular signaling. PathScan® Intracellular Signaling Array Kit (Cell Signaling Technology, USA, #7018) was used to detect changes in signaling molecules. All experiments followed the experimental process provided by CST and were repeated three times.

### Statistical analysis

Raw data were analyzed using one-way analysis of variance and Student’s t-test; statistical analysis was performed using SPSS 18.0 statistical software. All values in this study were calculated using mean ± standard deviation. P<0.05 has significant statistical significance.

### Data availability statement

All the data are available from the corresponding author upon reasonable request.

## Supplementary Material

Supplementary Figures
