# Supplementary material for: Pan-cancer analysis of NUDT21 and its effect on the proliferation of human head and neck squamous cell carcinoma
Source: Aging (Albany NY). 2024 Feb 12;16(4):3363–85. doi: 10.18632/aging.205539 (PMC10929839; doi:10.18632/aging.205539)
Supplement: Supplementary Figures [file aging-16-205539-s001.pdf]

SUPPLEMENTARY FIGURES

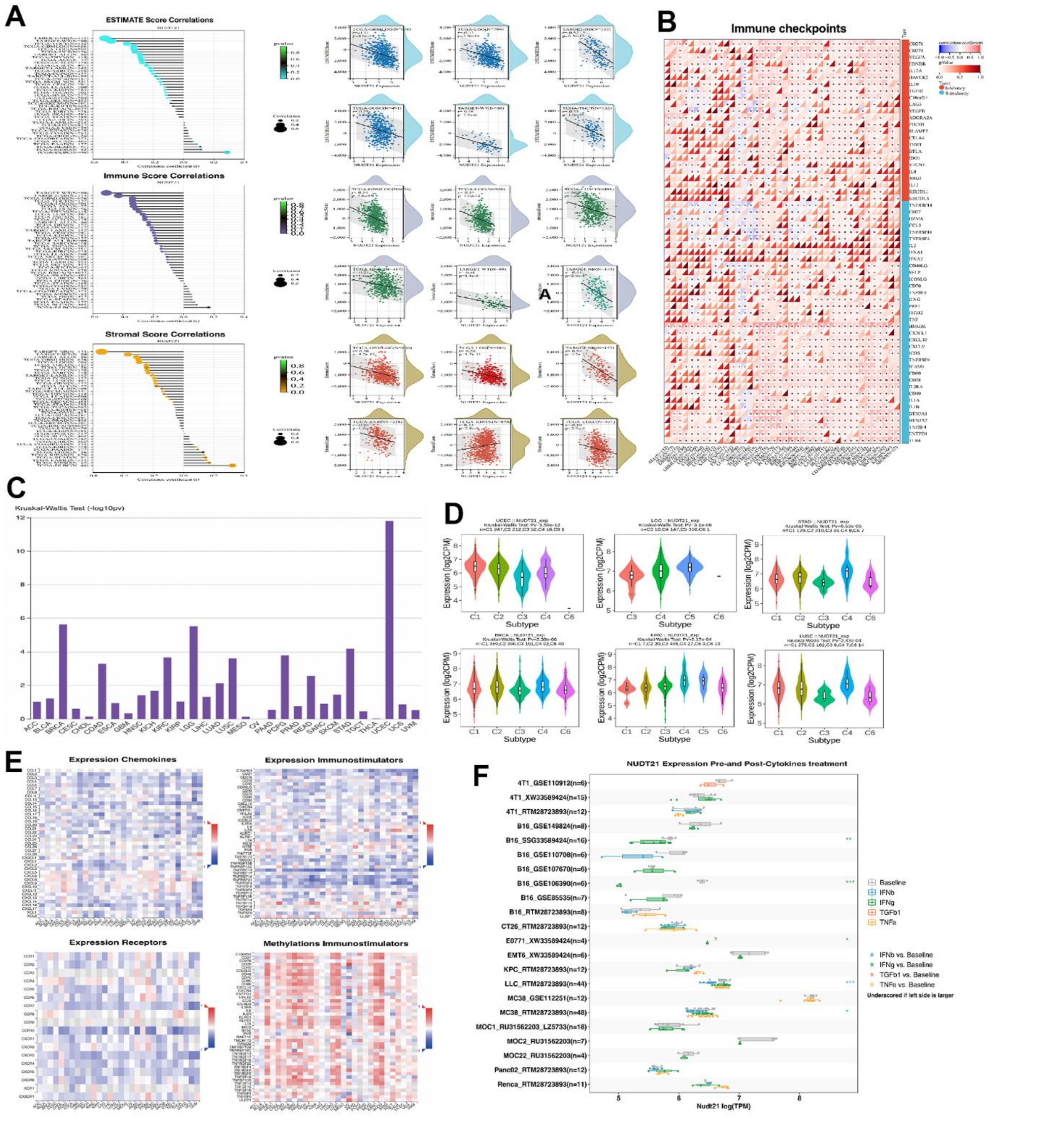

Supplementary Figure 1. NUDT21 interacts with immune infiltration and cytokines. (**A**) Histogram of correlations between NUDT21 and estimated score, immune score and stromal score (left), and cancer scatter plot showing the top 6 correlations for each score (right). (**B**) Heat map of the association between immune checkpoints and NUDT21 expression in pan-cancer. (**C**) Correlations between NUDT21 and immune subtypes using the TSIDB online tool. (**D**) Expression of NUDT21 in immune subtypes (C1, C2, C3, C4, C5, C6) in six cancers. (**E**) Correlation between NUDT21 expression and chemokines, receptors and immunostimulatory agents and heat map of immunostimulatory agents between NUDT21 promoter methylation levels. (**F**) Multi-box plots of NUDT21 expression in cancer cell lines before and after cytokine treatment were retrieved from the TISMO web tool, *, **, *** represent p <0.05, p < 0.01 and p < 0.001, respectively.

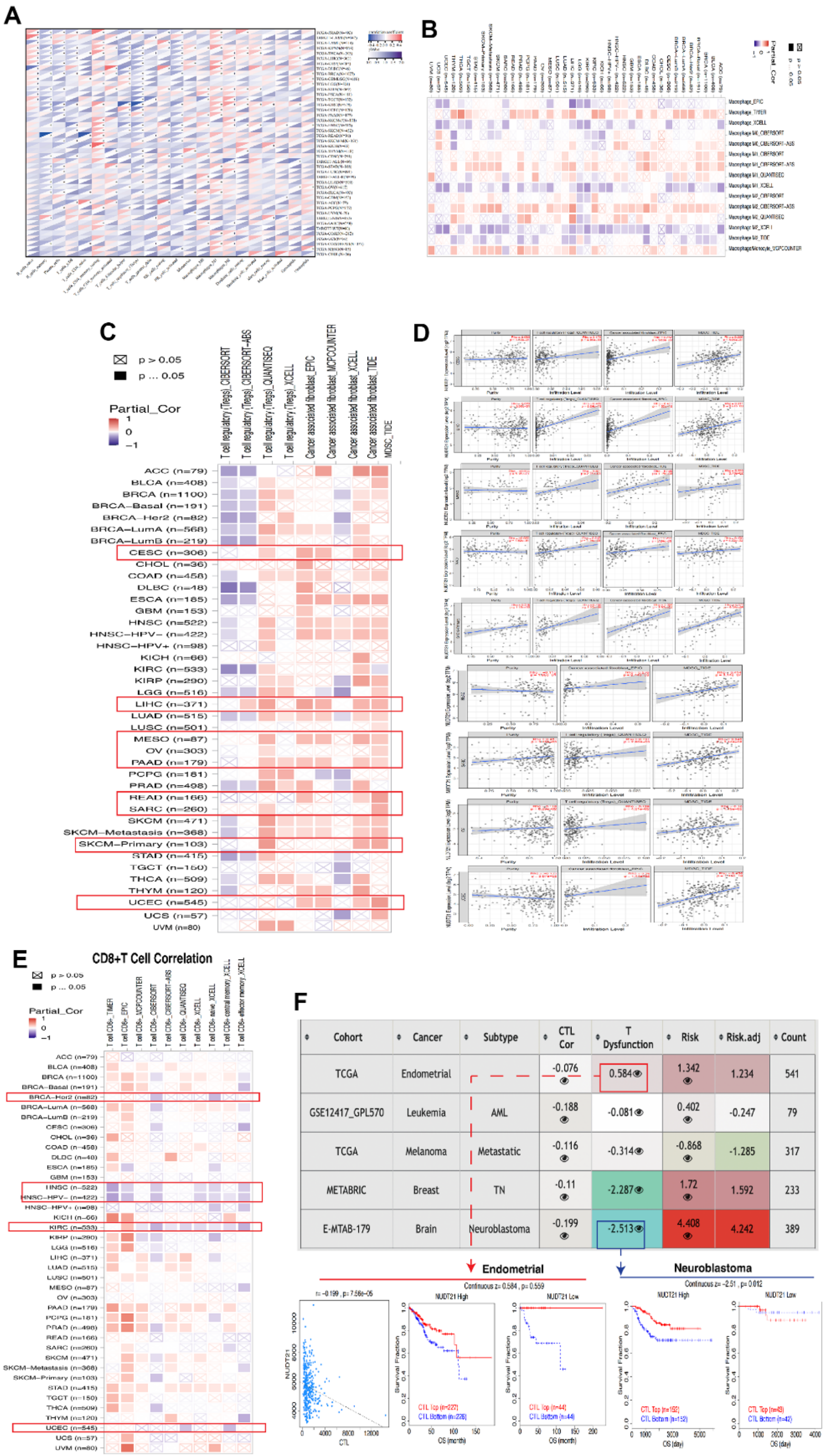

Supplementary Figure 2. NUDT21 is associated with M2 macrophages, Treg, CAFs, MDSC infiltration and CD8+ T cell suppression. (**A**) Cibersort classification calculation of 22 immune cell infiltrates in pancytosis. (**B**) Multiple algorithm calculations for M2 macrophage infiltration on TIMER 2.0. Partial_Cor indicates biased correlation. (**C**) Heat map of the association between NUDT21 levels and Treg, CAFs, and MDSC infiltration calculated using multiplex analysis on TIMER2.0. The red boxes highlight cancers with consistent trends in more than two cells. (**D**) Purity and purity-adjusted correlations between NUDT21 and Treg, CAFs, and MDSCs for nine cancers are highlighted in (**A**) and shown by scatter plots in TIMER 2.0. (**E**) Multiple algorithms calculated the correlation between NUDT21 and CD8+T cell infiltration. (**F**) Correlation of NUDT21 expression with CTL, CTL dysfunction and risk is shown in the table.
